# Health Problems in India

**Published:** 1921-09-10

**Authors:** 


### Health Problems in India.

### The Feminist Movement in Afghanistan.

A newspaper has recently been started in Cabul to give publicity to women's educational development and kindred subjects, including maternity and child welfare. The paper claims for women in Islam a much higher social position than is generally accorded to them by those unacquainted with Eastern life, and the very existence of this journal indicates a wave of interest in social problems which is apparently agitating the harems of Central Asia and will come as a surprise to many women in this country.

### Protection of Children in India.

The existing law governing the protection of children in India has for some time past been felt to be defective, and not in accordance with modern ideas on the subject. A Bill has just been introduced into the Bengal Legislative Council which follows the lines of the Children Act of 1908, but its operation is confined to boys.

It seeks to substitute educational treatment for penal measures, and provides for the establishment of reformatory and industrial schools by Government, and for the recognition of similar schools under private management. Broad distinctions between the two classes of schools are intended, the reformatory for the actual and the industrial schools for potential offenders.

### Vaccination in South India.

The Government of Madras has issued an Order calling the attention of Municipal Councils to the fact that in European countries vaccination is considered so important that it is always performed by qualified practitioners. The Order requests Council to discontinue the present practice of having the operation carried out by persons devoid of medical training, and suggests contracts with local private practitioners. The Order provides for the payment of the medical vaccinators, and institutes a proper system of records of results.

### The Census in Calcutta.

The Calcutta Corporation is not satisfied with the recent Census result, and in a letter to the Government of Bengal have asked to have an opportunity for checking the Census schedules. The preliminary figures show an increase of only 1 per cent. on 1911. During this decade there has been a great increase in the demand for houses in spite of extensive building activity, and these, together with marked increase in the consumption of water, point to a far greater increase of population than the figures indicate. The Corporation point out to the Government the importance of accurate Census returns in municipal life. If their check reveals any serious omissions the Government will propose a recount in March 1922.

